# Supplementary material for: Novel Ralstonia species from human infections: improved matrix-assisted laser desorption/ionization time-of-flight mass spectrometry-based identification and analysis of antimicrobial resistance patterns
Source: Microbiol Spectr. 2024 Apr 25;12(6):e04021-23. doi: 10.1128/spectrum.04021-23 (PMC11237764; doi:10.1128/spectrum.04021-23)
Supplement: Supplemental figures — Fig. S1 and S2. [file spectrum.04021-23-s0001.pdf]

Supplementary material

Novel *Ralstonia* species from human infections: improved matrix-assisted laser desorption/ionization time-of-flight mass spectrometry-based identification and analysis of antimicrobial resistance patterns

Stephanie Steyaert[a], Charlotte Peeters[a,b,#], Anneleen D. Wieme[a,c], Astrid Muyldermans[b,d,*], Kristof Vandoorslaer[d], Theodore Spilker[e], Ingrid Wybo[b,d], Denis Piérard[b,d], John J. LiPuma[e], and Peter Vandamme[a,b]

[a] Laboratory of Microbiology, Department of Biochemistry and Microbiology, Ghent University, Belgium.

[b] National Reference Center for *Burkholderia cepacia* complex, Belgium.

[c] BCCM/LMG Bacteria Collection, Laboratory of Microbiology, Department of Biochemistry and Microbiology, Ghent University, Belgium.

[d] Department of Microbiology and Infection Control, Vrije Universiteit Brussel (VUB), Universitair Ziekenhuis Brussel (UZ Brussel), Belgium.

[e] Department of Pediatrics, University of Michigan Medical School, Ann Arbor, Michigan, USA.

Running Head: Identification of *Ralstonia* in human infections

Address for correspondence: charlotte.peeters@ugent.be

*Present address: Department of Laboratory Medicine, Medical Microbiology, AZ Sint-Jan, Brugge, Belgium

Stephanie Steyaert and Charlotte Peeters contributed equally to this work. Author order was determined in order of increasing seniority.

Supplementary tables (excel file)

Supplementary table S1: Isolates included in the present study. CCUG, Culture Collection University of Göteborg; CCM, Czech Collection of Microorganisms; NCTC, National Collection of Type Cultures. NA, no information available. *, received as "*Pseudomonas thomasii*".

Supplementary table S2: Identification results. Columns E and F show the MALDI-TOF MS identification based on analysis of the Bruker ID score in reference to the Bruker (IVD V12.0, MSP-11758) database (column E) or the Bruker (IVD V12.0, MSP-11758) database combined with our *in-house* (RUO, MSP-5331) database (column F). Column B represents the final identification based on the analysis of the Bruker ID scores and the dereplication analyses.

Supplementary table S3: *Ralstonia* isolates present in the Bruker (IVD V12.0, MSP-11758) and *in-house* (RUO, MSP-5331) MALDI-TOF MS databases.

Supplementary table S4: Genome Taxonomy Database (GTDB) classification.

Supplementary table S5: Antimicrobial resistance and virulence genes.

Supplementary figures

Supplementary figure S1: Pairwise dDDH and ANI values between the 32 genomes from the present study and the genomes of the type strains of the 11 established *Ralstonia* species.

Supplementary figure S2: Phenotypic and biochemical characteristics of isolates in the present study. Test results are presented as positive (green), weakly positive (orange) or negative (red).

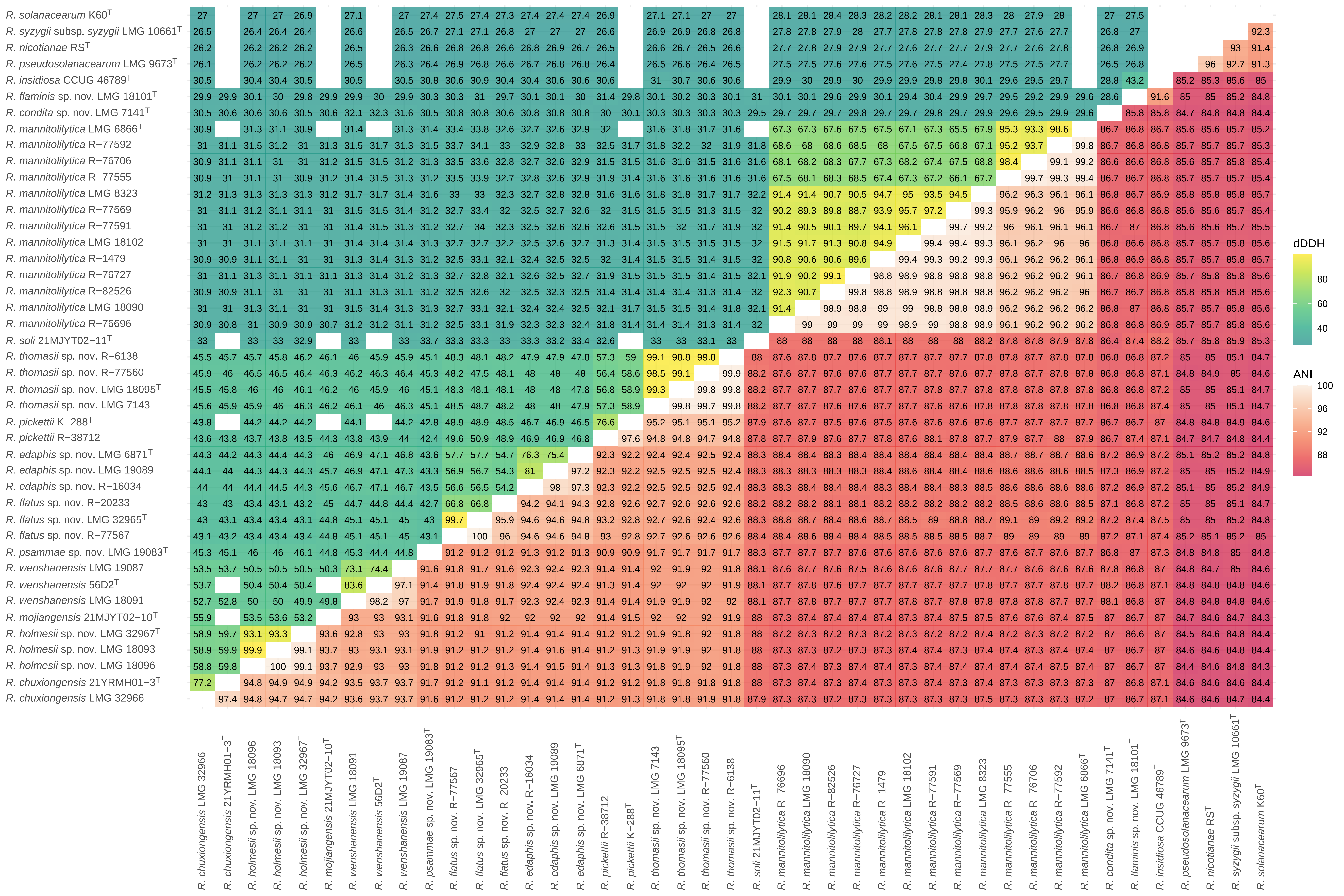

R. solanacearum K60T
R. syzygii subsp. syzygii LMG 10661T
R. nicotianae RST
R. pseudosolanacearum LMG 9673T
R. insidiosa CCUG 46789T
R. flaminis sp. nov. LMG 18101T
R. condita sp. nov. LMG 7141T
R. mannitolilytica LMG 6866T
R. mannitolilytica R−77592
R. mannitolilytica R−76706
R. mannitolilytica R−77555
R. mannitolilytica LMG 8323
R. mannitolilytica R−77569
R. mannitolilytica R−77591
R. mannitolilytica LMG 18102
R. mannitolilytica R−1479
R. mannitolilytica R−76727
R. mannitolilytica R−82526
R. mannitolilytica LMG 18090
R. mannitolilytica R−76696
R. soli 21MJYT02−11T
R. thomasii sp. nov. R−6138
R. thomasii sp. nov. R−77560
R. thomasii sp. nov. LMG 18095T
R. thomasii sp. nov. LMG 7143
R. pickettii K−288T
R. pickettii R−38712
R. edaphis sp. nov. LMG 6871T
R. edaphis sp. nov. LMG 19089
R. edaphis sp. nov. R−16034
R. flatus sp. nov. R−20233
R. flatus sp. nov. LMG 32965T
R. flatus sp. nov. R−77567
R. psammae sp. nov. LMG 19083T
R. wenshanensis LMG 19087
R. wenshanensis 56D2T
R. wenshanensis LMG 18091
R. mojiangensis 21MJYT02−10T
R. holmesii sp. nov. LMG 32967T
R. holmesii sp. nov. LMG 18093
R. holmesii sp. nov. LMG 18096
R. chuxiongensis 21YRMH01−3T
R. chuxiongensis LMG 32966
dDDH
80
60
40
ANI
100
96
92
88

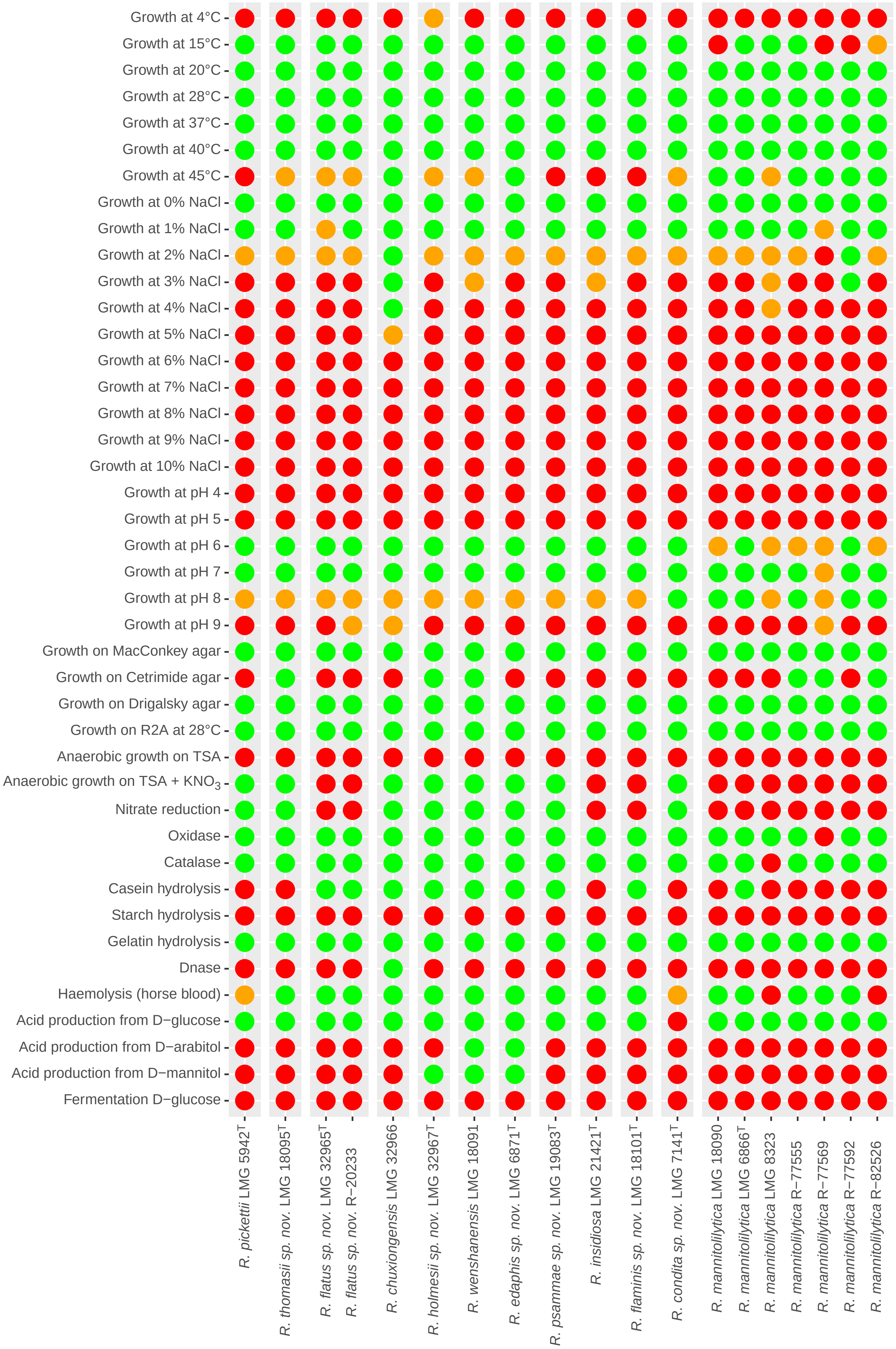
Growth at 4°C
Growth at 15°C
Growth at 20°C
Growth at 28°C
Growth at 37°C
Growth at 40°C
Growth at 45°C
Growth at 0% NaCl
Growth at 1% NaCl
Growth at 2% NaCl
Growth at 3% NaCl
Growth at 4% NaCl
Growth at 5% NaCl
Growth at 6% NaCl
Growth at 7% NaCl
Growth at 8% NaCl
Growth at 9% NaCl
Growth at 10% NaCl
Growth at pH 4
Growth at pH 5
Growth at pH 6
Growth at pH 7
Growth at pH 8
Growth at pH 9
Growth on MacConkey agar
Growth on Cetrimide agar
Growth on Drigalsky agar
Growth on R2A at 28°C
Anaerobic growth on TSA
Anaerobic growth on TSA + KNO_3
Nitrate reduction
Oxidase
Catalase
Casein hydrolysis
Starch hydrolysis
Gelatin hydrolysis
Dnase
Haemolysis (horse blood)
Acid production from D−glucose
Acid production from D−arabitol
Acid production from D−mannitol
Fermentation D−glucose
R. pickettii LMG 5942^T
R. thomasii sp. nov. LMG 18095^T
R. flatus sp. nov. LMG 32965^T
R. flatus sp. nov. R−20233
R. chuxiongensis LMG 32966
R. holmesii sp. nov. LMG 32967^T
R. wenshanensis LMG 18091
R. edaphis sp. nov. LMG 6871^T
R. psammae sp. nov. LMG 19083^T
R. insidiosa LMG 21421^T
R. flaminis sp. nov. LMG 18101^T
R. condita sp. nov. LMG 7141^T
R. mannitolilytica LMG 18090
R. mannitolilytica LMG 6866^T
R. mannitolilytica LMG 8323
R. mannitolilytica R−77555
R. mannitolilytica R−77569
R. mannitolilytica R−77592
R. mannitolilytica R−82526
